# SCIP: a self-paced summer coding program creates community and increases coding confidence

**DOI:** 10.1101/2022.12.27.521952

**Published:** 2022-12-27

**Authors:** Rochelle-Jan Reyes, Olivia Pham, Ryan Fergusson, Niquo Ceberio, Candace Clark, C Sarah Cohen, Megumi Fuse, Pleuni Pennings

**Affiliations:** 1 Biology Department, San Francisco State University, San Francisco, CA; 2 Department of Applied Physics and Material Sciences, Northern Arizona University, Flagstaff, AZ; 3 Bioinformatics and Systems Biology Graduate Program, UC San Diego, San Diego, CA; 4 Herbert Wertheim School of Public Health and Human Longevity Science, UC San Diego, San Diego, CA

## Abstract

In 2020, many students lost summer opportunities due to the COVID-19 pandemic. We wanted to offer students an opportunity to learn computational skills and be part of a community while stuck at home. Because the pandemic created an unexpected research and academic situation, it was unclear how to best support students to learn and build community online. We used lessons learned from literature and our own experience to design, run and test an online program for students called the Science Coding Immersion Program (SCIP). In our program, students worked in teams for 8 hours a week, with one participant as the team leader and Zoom host. Teams worked on an online R or Python class at their own pace with support on Slack from the organizing team. For motivation and career advice, we hosted a weekly webinar with guest speakers. We used pre- and post-program surveys to determine how different aspects of the program impacted students. We were able to recruit a large and diverse group of participants who were happy with the program, found community in their team, and improved their coding confidence. We hope that our work will inspire others to start their own version of SCIP.

## Introduction

In the summer of 2020, the COVID-19 pandemic caused many summer research programs and internships to be delayed or canceled. A survey in the United States reported that 44.5% of research opportunities for undergraduate students were canceled (Grineski, 2020), which left many students without the opportunity to advance their learning and career opportunities. Some undergraduate research institutions adjusted their summer programs to become virtual or created new online programs (Noriega 2020, Valerie Sloan 2020, Afghani 2021, Yowler *et al*. 2021), but this was not possible for many other programs.

At San Francisco State University (SF State) in-person learning was halted in March of 2020 and the SF State campus stayed almost entirely closed for summer research in biology and chemistry, which led to a huge loss of opportunity for our students. To provide our students a way to learn new skills despite the campus closure, we decided to create a new virtual program called the Science Coding Immersion Program (SCIP); this program was partly based on our previous experience with an on-campus summer coding program for non-computer science undergraduates (Pennings et al., 2020) and other related programs on our campus (Estrada et al., 2017; Reyes et al., 2022; Yoon et al., 2018). Our participants were primarily undergraduate and graduate students in Biology, Chemistry and Biochemistry. Our aim was to create a program where these students would learn coding skills and build community, during a time where many students lost their university community due to the stay-at-home and social distancing orders that prevented large gatherings.

Based on our aims, we had three major objectives for SCIP: (1) create a virtual workspace structure that could work while students were sheltered in place during the pandemic; (2) provide a space to build community for students; and (3) increase student confidence in coding as a scientific skill and enthusiasm for coding.

To address SCIP’s first objective, a structured *virtual* space was created for students to meet and communicate with other science students, using Zoom for team meetings and webinars, a Slack space for announcements and questions, and Google documents for instructions. The second objective of SCIP was addressed through our method for community building, in which participants work in teams for 8 hours a week; the team meetings were structured like study groups with a designated (volunteer) team leader who hosted the Zoom meetings and kept track of time. SCIP focused on community building primarily because of the known positive impact of community on learning. In the social theory of learning, learning communities can reduce attrition and advance learning (Wenger-Trayner et al., 2014). From this, building local communities within a scientific field was proposed to promote the learning of programming skills (Stevens et al., 2018). SCIP’s third objective was to increase the coding confidence of the participants who were mostly beginners in coding. This is important because many subfields in biology and (bio)chemistry depend on programming to analyze big data (Ouzounis & Valencia, 2003; Stres & Kronegger, 2019). We also realize that many of our students (majority female, many from groups that are underrepresented in the sciences) have not previously had opportunities to learn computing skills (Bruno & Lewis, 2021; Martin et al., 2015). There is therefore a particularly big need for our students to learn computational skills.

When we started the SCIP program, it was unclear whether it would work: Would students stay in a program that was ungraded? Would they learn if there was no live teaching? And would they find community in a 100% virtual program? Our study provides insight into SCIP’s program design and an evaluation on the effectiveness of the program on participant coding confidence. Analysis of the design and program effectiveness includes student evaluations of the program structure, the effectiveness of different forms of communication and self-perceived levels of confidence in coding.

## Methods

### Program Overview

There were three main considerations in developing the logistics of the online SCIP program: (1) offering multiple scheduling options to accommodate other summer activities of participants; (2) offering participants the choice to learn either Python or R; and (3) creating a system for successful communication between participants, team leaders and program coordinators.

The base structure of SCIP was adapted from the ten-step guide to support non-computer science undergraduates in learning how to code in a summer program (Pennings et al., 2020). For SCIP, the structure was modified to accommodate a larger number of participants with different schedules, last-minute planning for the organizing team and the participants, and a choice of different coding languages. Because the program was fully virtual, coordinators also created a strategy for communication among participants, team leaders and coordinators. In addition, we curated a reading list and offered a series of webinars to showcase possible career paths for scientists with coding skills. To address the participant needs and accommodations, coordinators incorporated Zoom meetings, staggered start dates (in 2020, but not 2021), two programming languages (a pilot study for ImageJ was additionally offered in 2021), and Slack workspaces, as well as curated readings, webinars.

#### Scheduling and Coding Languages

SCIP was designed to be flexible with participants’ summer schedules and interests. Potential conflicts included summer classes, other virtual summer programs, part-time jobs and family commitments. When the program was introduced rapidly in 2020 due to the short turnaround of program cancellations from the pandemic, the program included staggered start dates, which allowed coordinators to start with a small number of participants and to incorporate time for changes in running the program. It also allowed the large volume of registrants to join at a time that best suited their schedules. Therefore, participants chose their preferred Monday start date from 5 consecutive Mondays in 2020. The duration of the program varied from 8 weeks for the early start dates to 6 weeks for the later start dates. With sufficient time to advertise and organize as such in 2021, the program established one start date for all participants, with the same duration for the program. In 2021, all participants therefore started on the first Monday of June for a 6-week program.

Along with program start dates, participants were also asked in the registration to indicate their (1) available times (e.g., morning, afternoon, evening), (2) programming language of choice, and (3) experience level with coding prior to the program. Each participant was also asked to indicate their willingness to be a team leader. To be a team leader, interested participants were expected to have leadership experience of any kind, but they did not need to have previous coding experience. Coordinators then created teams of 4–8 participants and 1 team leader based on these factors to ensure participants’ availability, as well as similar interests and experience.

Each team worked independently on a course that was chosen by the program coordinators (Supplemental Table S1). The courses we offered in 2020 were the free beginner Udacity courses of *Data Analysis with R* (Udacity: Data Analysis with R, n.d.) and *Introduction to Python Programming* (Udacity: Introduction to Python, n.d.). In addition to R and Python, we added an image analysis option in 2021, using ImageJ as a learning platform. This was a small part of the program with just two image analysis teams, mostly with students who were slated to participate in another workshop at the end of the summer offered by the NSF-supported Center for Cellular Construction. With these selected courses, participants were encouraged to work at their own pace and not rush to finish the course. For those who were interested in more structure, we provided a timeline with suggested course sections to go through per week but with the emphasis that participants did not have to follow it if it did not support their learning.

#### Online Communication Platforms

Slack was used as the primary communication tool between the SCIP coordinators and the participants (Tuhkala & Kärkkäinen, 2018; Vazquez et al., 2020). To assure efficient communication, all participants were required to join our Slack workspace before starting the program. The purpose of the platform was to provide a space for participants to communicate with their teams, SCIP coordinators, and any other participant in the program. General channels were accessible to everyone for webinar reminders and general announcements, and a question forum was available for participants to ask questions and share tips. In addition, a separate channel for each team was created to discuss coding and meeting topics outside of their team meetings, if necessary. These team channels only included members of the team and no coordinators in order to provide a space for team participants to freely discuss questions, answers, and messages.

Zoom was used for all team meetings and for the webinars. Teams used Zoom to meet virtually with their group and work on their Python or R courses four times a week for two hours. At the start of the program, team leaders were tasked to create a Zoom link and facilitate team meetings. Weekly webinars were also held through Zoom to provide participants insight on research and career options within the Biology and Chemistry/Biochemistry fields that use coding skills. Guest speakers ranged from professors to SF State alumni in graduate programs and/or biotechnology jobs.

#### Program Learning Structure

What sets SCIP apart from other online learning structures is the *immersion* aspect of the program, which allows participants to *learn both as part of a community and individually*. In SCIP, teams meet for 8 hours a week and about 4 of those hours are designated as “quiet working time”, which was based on the “Shut Up and Write” idea that is used in writing groups (Mewburn et al., 2012). Participants were encouraged to work on their online course during the “quiet working time” in their team meetings only, and not outside of the team meetings. Therefore, participants learned new skills while they were in Zoom meetings with each other during the reserved meeting time, rather than working on it in their own time. This structure allowed for participants to work on their course individually, but have a community and support system in which they can reach out to when in need of support and guidance. Knowing that learning computer programming can be very frustrating, we structured our program this way so that participants would never struggle with coding tasks on their own (Beaubouef & Mason, 2005; Chase & Okie, 2000).

Zoom meetings were hosted and facilitated by team leaders who were also peers. Studies have shown that peer-led teams have a positive impact on participants’ performance, retention, and attitude (Batten & Ross, 2021; Quitadamo et al., 2009; Tien et al., 2002; Trujillo et al., 2015). Most of our team leaders had little-to-no coding experience (survey data not shown). To better align with the coding experiences of the participants, team leaders were assigned to teams with similar coding experience and learned the same material as everyone else in the team. Additionally, SCIP coordinators created a schedule for them to use in the meetings with very detailed information for the first few days and less detailed later on (Supplemental Document S3). The material for team leaders included sample emails for them to send to their team, and checklists for what they were expected to do each week (Supplemental Table S2). The purpose of providing these materials to team leaders was to reduce any extra time preparing for meetings and increase potential for successful team meetings.

Team leaders were instructed to make sure that each Zoom meeting consisted of time to talk (check-ins, problem solving, brainstorming etc.) and time to study (read, view course videos, do coding exercises) while the Zoom meeting was muted (Supplemental Table S2). Teams were not restricted to the suggested format and were free to create their own schedule as long as the following topics were covered every Zoom meeting: (1) brief check-ins to share how they were doing or how they felt about coding that day, (2) weekly suggested reading and discussion, (3) a total of 1 hour of quiet working time or “Shut Up and Code” time per session, (4) collection of questions to answer as a team or post on Slack and (5) reflections.

The program provided a curated list of suggested readings, inspired by the idea of scientist spotlights (Schinske et al., 2016; SFSU SEPAL, n.d.; Yonas et al., 2020). Scientist spotlights are curriculum supplements that let students learn about counter-stereotypical scientists. In addition to scientist spotlights, the curated reading list also included blog posts and short articles about computer programming. The goal of these readings was to let SCIP participants learn about both frustrations and successes from others who have come before them. In order to achieve this specific goal, we included short interviews with SF State alums who have dealt with frustration as they were learning to code (normalizing frustration) and how they have overcome difficulties (e.g., by asking others for help) and found success (e.g., a job or admission to a graduate program) (Supplemental Document 3).

### Analytic Methods

Evaluation of program effectiveness was analyzed through data from two participant surveys: the pre- and post-program assessments. Participation in the survey was voluntary, and all participant survey responses were collected anonymously. Because group start dates were staggered in 2020, the pre-assessment survey was given after the last offered start date of the program and the post-assessment survey was given at the end of the entire program. For 2021, pre-assessment was given on the first day of the program since all participants started on the same day and post-assessment was sent out at the end of the program.

Pre- and post-program surveys were administered via Google forms, to obtain evaluation of the program as well as its components, through the perspective of the participants (Supplemental Document S1 and S2). In particular, we evaluated the participants’ overall confidence in coding before and after participating in SCIP, while also collecting demographic and participation data. *Numerical rating scale questions*, in the form of Likert-type data, were asked in both assessments to evaluate confidence in coding as well as effectiveness of the program design. In the post-assessment, some questions were *open-ended* to elicit broader comments or suggestions about the SCIP program. The study was IRB exempt. Data and code for data analysis are available in a GitHub repository (Reyes & Pham, 2022).

To compare self-reported coding confidence before and after the summer program, we used a chi-squared test. To analyze answers to the open-ended question “*What is one thing you liked about the program?*”, we created a word cloud along with a frequency measurement as well as an informal coding of the answers to count how often certain aspects of the program were mentioned. All statistical and descriptive analyses were done in R Statistical Software (R Core Team, 2021).

## Results

### Demographics

**Figure 1.**
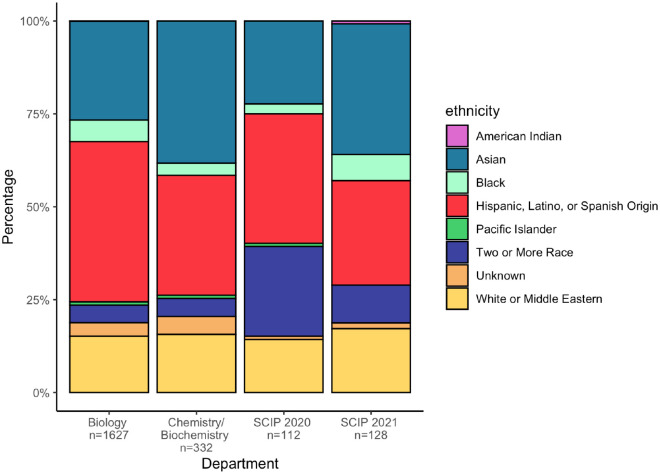
Race and Ethnicity of participants in SCIP mirrored the demographics of the Biology and Chemistry/Biochemistry departments. Participants who identified with more than one race and/or ethnicity were counted within the “Two or More Race” category. Spring 2020 data for Biology and Chemistry/Biochemistry were taken from SF State Institutional Research (www.ir.sfsu.edu).

Based on the pre-assessment survey, participants’ self-identified race and ethnicity mirrored SF State enrollment statistics in the Biology and Chemistry/Biochemistry departments for Spring 2020 (Office of Institutional Research, SF State: https://ir.sfsu.edu/; Figure 1). For both cohorts, the biggest groups were of Hispanic/Latinx (34.8 and 28.1% for 2020 and 2021 respectively) and Asian (22.3 and 35.2% for 2020 and 2021 respectively) descent, followed by White and mixed-race. Of the students enrolled in SF State Biology and Chemistry/Biochemistry departments for spring 2020 data, 41.3% identified as Hispanic or Latinx descent.

In terms of gender, for 2020 and 2021 respectively, 60.8 and 59% of participants identified as women, 2.5 and 5.2% as non-binary, trans or gender-non-conforming, and 36.7 and 35% as men. Amongst biology students at SFSU, 70% identify as women and 29.8% as men, less than 1% identify as non-binary. The SCIP program therefore has a high percentage of women, but not as high as the percentage in the biology department.

In the summer of 2020, just over half (54.5%) of the participants were undergraduate students, 35.7% were master’s students and the remaining participants comprised of post-baccs, faculty and staff (Supplemental Figure S1). In the summer of 2021, undergraduate and master’s student counts were similar at 54.7% and 27.3%, respectively (Supplemental Figure S1). Most participants were Biology and Chemistry/Biochemistry students (Supplemental Figure S1).

### Program Evaluation

#### Online Communication Platforms and Effectiveness

**Figure 2.**
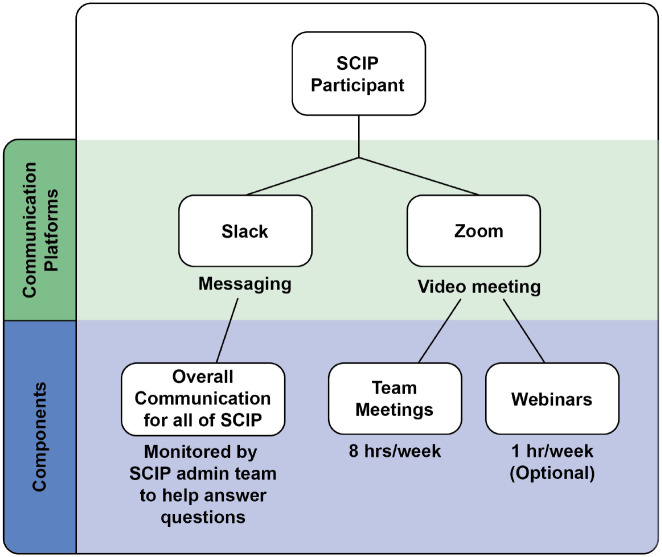
Communication platforms adopted in the program.

In order to streamline communication, two communication platforms - *Slack* and *Zoom* - were chosen and used throughout the program (Figure 2). These platforms were chosen because they were free to use and most of the participants had prior experience with the use of these platforms at SF State. For example, SF State adopted Zoom conferencing services (Zoom Video Communications Inc., 2016) for virtual classes during the pandemic, and Slack (Slack Technologies) was integrated into some research labs and used by the majority of students funded by public (NIH and NSF) and private (Genentech Foundation) fellowships through the Student Enrichment Opportunities Office (https://seo.sfsu.edu/), as well as by the Department of Biology.

**Figure 3.**
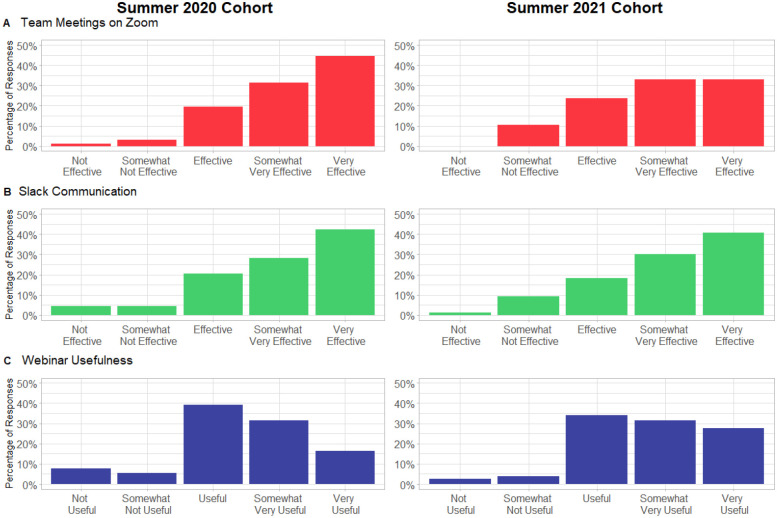
Survey-reported effectiveness of the use of (A) Zoom for team meetings, and (B) Slack workspace and (C) webinars, as media to communicate with team members as well as other SCIP participants and coordinators.

According to our post-survey assessment, 95.7% (88/92) of our participants felt that using the Zoom meetings with their teams were “Effective”, “Somewhat Very Effective” or “Very Effective” in 2020, and 89.5% in 2021 (Figure 3A). For Slack, 91.3% (84/92) of the participants felt that the Slack workspace was “Effective” to “Very Effective” in 2020 and 89.5% in 2021 (Figure 3B). Webinars likewise were perceived as “Effective” to “Very Effective” by 87.0% of respondents in 2020 and 93.4% in 2021 (Figure 3C).

#### Participation and Retention Rates (Supplemental Table S3)

Participation and retention rates were assessed in three different ways within the program. First, we counted the number of students who let us know that they left the program. Based on these numbers, 95.9% and 92% of participants retained throughout the program in 2020 and 2021, respectively. Secondly, retention of participants in the program was assessed through the completion rate of the pre-assessment and post-assessment surveys. The survey completion rate for the pre-assessment was 76.2% in 2020 (112 out of 147 participants) and 92.8% for 2021 (128 out of 138) in 2021. Survey completion rate for the post-assessment survey was 62.6% (92 out of 112) for 2020 and 55.1% (76 out of 128) for 2021. Third, we collected Zoom team photos, which were taken in the last two weeks of the program, and referenced them as a form of retention evaluation. Referencing these photos and accounting for the 7 teams (40 participants) who did not submit a team photo, 74.8% of the (80 out of 107) participants were present in the team photo in 2020. In 2021, we received photos from all teams and 81.2% (112 out of 138) of participants were present in their team photo. We believe that the team photos from the last week of the program are a good measure of retention, which means that more than 80% of our participants stayed for the entire program.

Additionally, to provide an encompassing overview of general satisfaction with the program, participants were asked in both 2020 and 2021 whether they would recommend SCIP to others. The question was: “*Would you recommend SCIP to others?*”. Nearly all of participants would indeed recommend SCIP to others (99% 2020 and 97% 2021) (Supplemental Figure S2B/C).

### Program Learning Effectiveness

#### Coding Confidence

We were interested in determining participants’ coding experience prior to SCIP and participants’ coding confidence before and after SCIP. In both cohorts, the majority of the participants assessed had no or little experience with coding: 50.7% and 50.8% had no experience in coding, and 18.7% and 31.7% had less than 1 semester of experience, in 2020 and 2021 respectively (Supplemental Figure 3). To find out if SCIP had an impact on participants’ coding confidence, we used the pre-assessment and post-assessment surveys to find out how confident participants felt about their coding skills on a numerical rating scale from 1 through 5.

**Figure 4.**
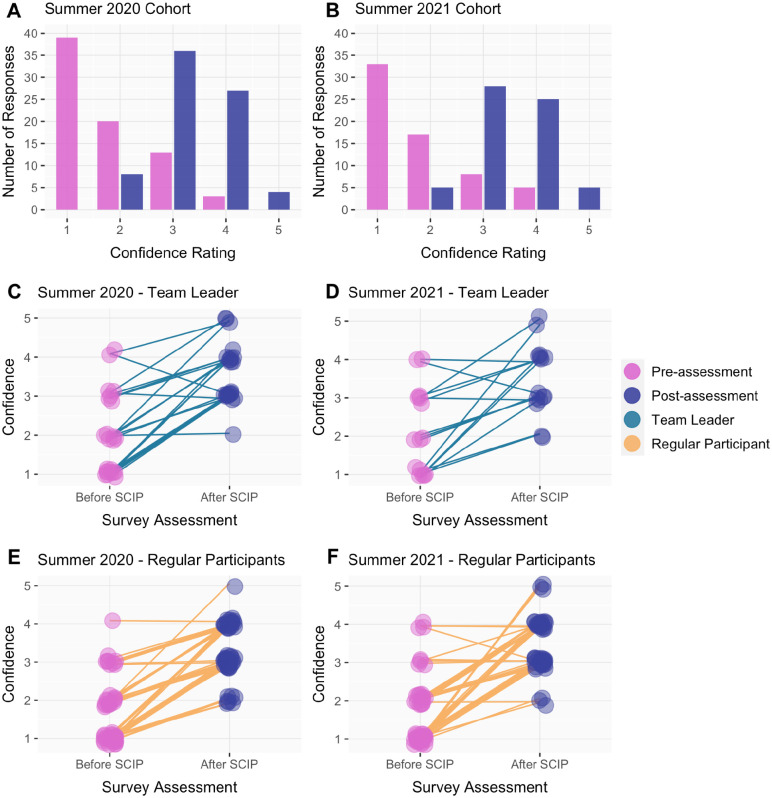
Coding confidence of 75 participants with paired survey responses for pre- and post-assessments in 2020 and 63 participants in 2021. (A/B) The number of responses per numerical rating based on the question “Before SCIP/After SCIP, how confident did you feel in your coding skills?” for 2020 and 2021, respectively; (C/D) Paired survey responses of participants who identified as team leaders. Each line is an individual participant indicating coding confidence prior to (pink) and after (purple) the completion of SCIP for each year; (E/F) Paired survey responses of participants who were regular participants. Each line is an individual participant indicating coding confidence prior to (pink) and after (purple) the completion of SCIP for each year.

Here, we report only on the participants for which we could pair the pre-assessment and post-assessment surveys. In 2020, we were able to pair 75 pre- and post-assessments. In 2021, we were able to pair 63 pre- and post-assessments. 7 paired survey observations from 2021 were excluded because the participants took part in the Image Processing groups that did not learn coding skills. For SCIP Summer 2020 and Summer 2021, median coding confidence from all participants increased significantly in both cohorts. The median coding confidence for all participants increased 2 points - from 1 in the pre-assessment to 3 in the post-assessment - with a p-value of 4.32e-16 (df=4) and n = 75 for Summer 2020 and a p-value of 3.71e-14 (df=4) and n = 63 for Summer 2021. Since the p-values are less than 0.05, we can infer that the increase in coding confidence for all participants (team leader and regular participant) was significant (Figure 4A/B)

For team leaders, the median coding confidence for both cohorts increased by 1 point - from 2 in the pre-assessment to 3 in the post-assessment - with a p-value of 1.65e-04 (df = 4) and n = 23 for Summer 2020 and a p-value of 0.04 (df=4) and n = 15 for Summer 2021. Based on the p-value, we can infer that team leaders had a significant increase in median coding confidence. Furthermore, we analyzed regular participants’ median coding confidence. The median coding confidence for regular participants increased 2 points in both cohorts, from 1 in the pre-assessment to 3 in the post-assessment - with a p-value of 1.07e-11 (df=4) and n = 52 for Summer 2020 and a p-value of 1.03e-12 (df = 4) with an n = 48 for Summer 2021 (Figure 4C/D/E/F). All in all, we found that the SCIP program increased self-assessed coding confidence for team leaders and regular participants.

#### Sense of Community

**Figure 5.**
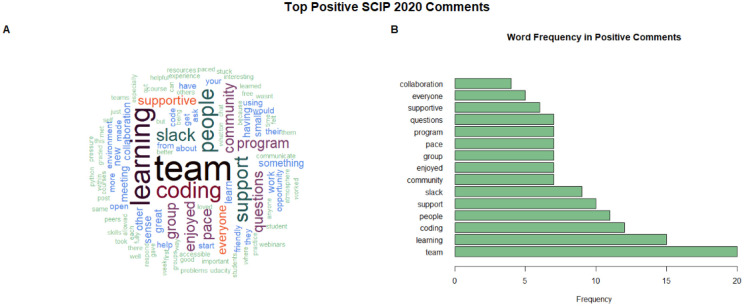

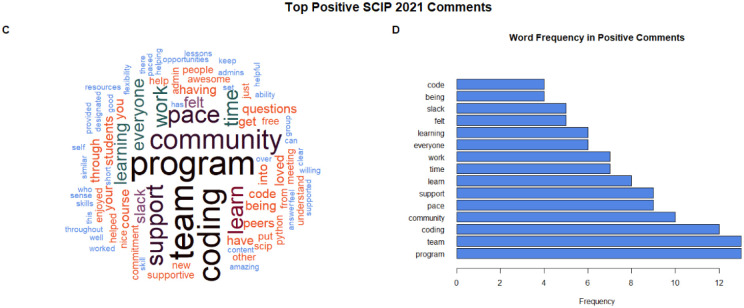
Words mentioned by participants in the post-assessment survey with the prompt, ““What is one thing you liked about the program?”. Words were taken from the open-ended prompt and consolidated into a count of the words used, from highest count to lowest count. (5A/C) Word Cloud of word frequency of the question with the most frequently used words in larger font size and location, in 2020 and 2021, respectively. (5B/D) Bar plot of the frequency of words used in the question, in 2020 and 2021, respectively.

We analyzed the answers to an open-ended question “*What is one thing you liked about the program?*” to determine whether our aim of creating a community had been achieved. Survey responses were first split into a list of words, and then the frequency of the words was determined. Common conversational filler words such as “the”, “a”, and “is” were omitted. We created a word cloud to illustrate the most commonly utilized words in the open-ended prompt (Figure 5A/C). We then identified the top 15 most frequently stated words based on survey responses. Five of the top 15 frequently stated words associated with positive aspects of the program were “team”, “people”, “community”, “group”, and “collaboration”, with “team” being the most frequently used word in 2020 (Figure. 5A and B). In 2021, positive aspects of the program continued to involve similar words to those in 2020, such as “team” and “community” (Figure 5C and D).

We also carried out an informal thematic coding of the responses to this question. We read all responses (92 in 2020 and 83 in 2021) and recorded whether the response mentioned any of the following: the team aspect of the program, the community aspect, support, not graded, learning, own pace, skills. In 2020, 23% of the respondents wrote something positive about the team aspect (e.g., one respondent wrote : “*I liked that we had our own small teams, it made learning code less intimidating*.” In 2021, 11% of the respondents reported something positive about the team aspect of the program. Here we also found that the word “team” was used in the combination “admin team”, which is how we referred to the students and professors who ran the program and answered participants’ questions. For example, one respondent wrote in 2021: “*I felt incredibly supported from the beginning! We were set up for success! Amazing job by the SCIP Admin team! Loved the program”*. “Support” was mentioned in 13% and 23% of the responses in 2020 and 2021 respectively, though in some cases, it was not entirely clear which aspect of the program gave the participant support. For example, one respondent in 2021 wrote: *“I got to learn coding in a supportive environment*.”, where it is not clear whether they meant their team, the admin team or the entire program. “Community” was mentioned by 18% and 20% of the responses in 2020 and 2021 respectively. One respondent in 2020 wrote “*A community to talk to virtually during quarantine.”* Others simply wrote: “*The community*” or “*Meeting new people*”.

## Discussion

We have described the SCIP coding program for biology and (bio)chemistry students, which we started in response to the pandemic in 2020. We have described how we organized the program, who we were able to recruit and how it created a community, and how it impacted student’s coding confidence. While we started SCIP because of the lockdown in 2020, we have now learned that there is a lot of interest even when there is no lockdown. For students with summer jobs or family obligations, it is often hard to commit to full time internships or summer programs, yet these students can more easily join an online and part time summer program like SCIP.

### Online Learning Program Structure was Effective for Student Learning

Overall, participants seemed to generally approve of SCIP’s program design, and we can infer that our program design can be used to provide participants a good opportunity to learn a skill for their career and build community. Participants generally approved of the Zoom meetings and Slack environment. Slack data shows that around 120 participants were active on Slack every week and around 80 posted each week (out of 147 and 138 total participants in 2020 and 2021 respectively), so the Slack environment was used well. We found that participants were somewhat less happy with the webinars, although it is not clear if that was because they didn’t participate or because they didn’t find the content useful. Future iterations of the program will look into this.

#### Participation and retention rates were strong

We used several different methods to estimate retention rates for the program and believe that the team photos from the last week of the program are a good measure of retention, which means that more than 80% of our participants stayed for the entire program. While we will always strive for higher retention rates, we feel that 80% retention is good for a program that is entirely voluntary and doesn’t give academic credit. If the students stay it is because they enjoy the program and/or feel that it is useful.

### Coding Immersion Creates a Scientific Community

One goal of SCIP was to help students feel part of a community during a period when they were not on campus. Although labs began to open up by 2021, live classes at SF State were still very limited and a significant amount of time was spent with remote learning. We gauged the scientific community by (1) analyzing who joined SCIP, (2) participation and retention in the SCIP program and (3) the level of satisfaction in the program, including plans to continue learning coding and (4) an analysis of how students felt about the program using open-ended questions on the post-assessment survey.

#### SCIP participant pool mirrored SF State Biology demographics

We recruited students mainly from the Biology and Chemistry/Biochemistry departments at San Francisco State University. We included messages in the department newsletters, sent emails to students in our classrooms (e.g., Genetics), and to all students in our research training programs (e.g., MARC, RISE). It was made clear that the program was free and students would not earn academic credit for it. All students majoring in Biology, Chemistry, or Biochemistry who applied were accepted to the program. We also accepted a few students from other schools, some alums from our own departments and several interested faculty and staff.

Latinx and Black students are often underrepresented in computing fields. Therefore, we wanted to determine whether Latinx and Black students are represented in this program as well. Our program had a somewhat lower number of Latinx students compared to the departments we recruited from. This could be in part due to the fact that we allowed participants to check multiple ethnicities, in which case we counted them as mixed-race. Furthermore, although the Black student population is not large in general at SF State, our demographics mirrored those in the Departments of Biology and Chemistry/Biochemistry, and increased in 2021. Based on our findings, we see that Latinx and Black student representation in our program mostly matches the proportion of students from the departments we recruit from.

#### Participants felt a sense of community by the end of the program

To get a deeper understanding of what part of our program’s learning structure worked well for participants, we offered survey respondents an open-ended prompt to discuss what they enjoyed about the program. The wording of the prompt was as follows: “*What is one thing you liked about the program?*” and we found that participants especially appreciated the team-based and community aspects of the program.

### Coding Confidence increased for most participants

Most of our participants had little or no coding experience, and we were interested to determine if our program increased their coding confidence. We found that the median coding confidence for participants (team leaders, participants only, and combined values) increased by 1–2 points. These results showed that SCIP participation increased individual levels of coding confidence, providing evidence for the effectiveness of a virtual learning program for science students learning programming in R and Python.

Assessment of confidence in coding was also analyzed through interest in taking a coding class in the future through the survey responses. Of the participants who completed a post-program survey, over 75% of individuals were planning or were considering taking a coding class in the future. Even of those with no prior experience, more than two-thirds of participants responded that, after the program, they were considering or planning to take a class with a coding component in order to further their coding experience (Supplemental Figure S4).

Our program and assessment have some limitations. In 2020, the “pre-program” survey was actually done mid-program. This could have impacted the coding confidence results, but they were actually very similar in both years, which suggests that the results were valid even in 2020. In addition, because the program was not graded and participants did not submit any work to provide documentation for learned materials, we cannot easily judge how much they learned. However, we did ask participants how far they progressed in the online class that they were assigned. We found that in 2021 42% of participants who did the post-program survey indicated they finished the entire class and another 42% indicated that they finished three-quarters of the class. In 2020 35.2% indicated they finished the entire class and 46% indicated that they finished three-quarters of the class. All others completed one quarter or half of the class. Future iterations of this program may include more concrete survey questions to determine how much students have learned.

## Conclusion

The impact of the pandemic on our student population included loss of income, loss of family members, and loss of research and internship experiences. We developed a virtual program that provided students with opportunities to learn a new skill and acquire confidence in this skill, while also building a community from behind a computer screen. Two faculty and three part-time staff members managed over 130 participants in this virtual environment in 2020 and 2021. Retention was high over the 6–8 week period and participant feedback was very positive. Coding confidence increased in nearly all participants, and the participants appreciated the community and support they found in the self-paced program.

Participant comments in the open-ended questions suggested that the community was an important part of the learning environment: *“I liked the fact that my team was open to communicating in and out of zoom. I felt comfortable talking to them and asking them questions”, “I liked that we all had fun doing it, struggling, teaching each other”* and “*It was a safe supportive environment: everyone was really nice. I liked getting to know my team members*”.

It has become clear that the challenges of shifting from in-person learning to online learning, have been far greater than just technical adjustments. Studies worldwide have shown that the lockdown associated with the COVID-19 pandemic has had many impacts on student mental health, including changes in eating behaviors, sleep, anxiety, depression and suicidal thoughts (Flaudias et al., 2020; Kaparounaki et al., 2020; Son et al., 2020; Wathelet et al., 2020; Yang & Ma, 2020). Given the role that isolation has on mental health in general and its particular impact on students during the pandemic, through isolation from social networks, a lack of interaction and emotional support, as well as physical isolation (Elmer et al., 2020), it would be of interest to assess the impacts of a program like SCIP on the mental health of students.

Finally, as science becomes more interdisciplinary, coding and data science are clearly taking a prominent role in biological and (bio)chemistry research. The increase in confidence gained by our participants, many of them from historically underserved groups, could lead to more racial and gender diversity in fields not typically diverse, but where these computational skills are particularly important - such as neuroscience, computational biology, evolutionary biology and ecology. Taking advantage of the successes of our virtual program, we aim to offer this program to more students and also hope that other campuses will learn from our program. The SCIP method can be used within specific research fields, to draw students from across the country with this free format.

Our future goals are to create new online courses with diverse representation amongst the course instructors to offer as part of future SCIP programs, as a mechanism to increase coding skills amongst our students and to increase diversity in computational fields.

## Supplementary Material

Supplement 1

Supplement 2

Supplement 3

Supplement 4
